# Neuroprotective Roles of Lauric Acid and Resveratrol: Shared Benefits in Neuroinflammation and Anxiety, Distinct Effects on Memory Enhancement

**DOI:** 10.1002/fsn3.4520

**Published:** 2024-10-23

**Authors:** Betul Kisioglu, Esra Onal, Derya Karabulut, Ilyas Onbasilar, Asli Akyol

**Affiliations:** ^1^ Department of Nutrition and Dietetics, Faculty of Health Sciences Hacettepe University Ankara Turkey; ^2^ Department of Histology and Embryology, Faculty of Medicine Erciyes University Kayseri Turkey; ^3^ Transgenic Animal Technologies Research and Application Center Hacettepe University Ankara Turkey

**Keywords:** CD36, fructose, high‐fat diet, lauric acid, neuroinflammation, resveratrol

## Abstract

Neuroinflammation can be triggered by a high‐fat/high‐fructose diet (HFFD), and CD36 may be an underlying mechanism. Lauric acid (LA), the major fatty acid in coconut oil, and resveratrol, the plant‐based polyphenolic compound, may exert anti‐inflammatory effects. Therefore, this study investigated the possible effects of LA and resveratrol on diet‐induced neuroinflammation and CD36. Healthy male C57BL/6 mice (8 weeks of age, *n* = 31) were fed a control diet (10%kcal fat) or diets containing high fat (60%kcal fat) and fructose (5% w/v fructose drinking water) for 6 weeks, *ad libitum*. Supplemented to the HFFD, mice daily received resveratrol (7.5 mg/kg) (HFFD‐RSV) or LA (750 mg/kg) (HFFD‐LA). At the end of the study, HFFD resulted in anxiety‐like behavior, reduced locomotor activity, neuroinflammation (increased brain GFAP, IL‐6, MCP‐1, IFN‐γ, TNF‐α), and systemic inflammation (increased plasma GFAP, IFN‐γ, TNF‐α, IL‐12p70, reduced plasma IL‐10). HFFD‐RSV and HFFD‐LA alleviated HFFD‐induced anxiety‐like behavior, neuroinflammation, and systemic inflammation. HFFD‐LA improved memory. Brain and plasma CD36 levels were increased by HFFD and reduced by HFFD‐RSV or HFFD‐LA. Dietary resveratrol and LA intake may alleviate HFFD‐induced neuroinflammation, systemic inflammation, and anxiety‐like behavior and improve memory, as CD36 may be an underlying mechanism.

## Graphical abstract

In this study a high‐fat/high‐fructose diet induced systemic inflammation, neuroinflammation, and associated behavioral changes in mice. Further, dietary resveratrol or lauric acid intake has the potential to alleviate the damaging effects induced by a high‐fat/high‐fructose diet. Dietary lauric acid may improve recognition memory. As a nutrient‐sensitive biomarker, CD36 may be an underlying mechanism in diet‐induced neuroinflammation.

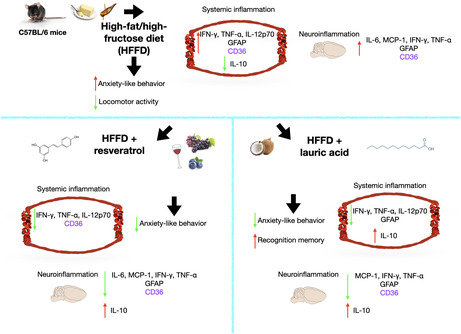

**Abbreviations**
CD36: cluster of differentiation 36
CO: coconut oil
DI: discrimination index
GFAP: glial fibrillary acidic protein
HFFD: high‐fat/high‐fructose diet
LA: lauric acid
MCFA: medium‐chain fatty acid
NOR: novel object recognition
NSFT: novelty suppressed feeding test
OFT: open field test
PCA: principal component analysis
RSV: resveratrol
SFA: saturated fatty acid

## 1 Introduction

World Health Organization (WHO) recommends focusing on nutritional interventions as modifiable risk factors to prevent neurodegenerative diseases (World Health Organization 2019). The daily diet is essential for normal brain function as several nutrients can cross the blood–brain barrier and regulate neurologic networks (McGrattan et al. 2019). However, the effect of diet and nutrients on neuroinflammation, the hallmark of neurodegenerative diseases (Gilhus and Deuschl 2019), needs to be better investigated. Neuroinflammation in rodents was reported as higher concentrations of neuroinflammatory markers (IL‐1, IL‐6, TNF‐α) in the brain (Alghamdi 2021; Li et al. 2019; Nakandakari et al. 2019) and serum (Ho et al. 2020) along with poorer performance in memory (Alghamdi 2021) and increased anxiety‐like behavior (Kloster et al. 2021) as a result of high fat or high fructose intake. Recent studies on C57BL/6 mice have shown that high‐fat/high‐fructose diets increase brain TNF‐α (Dong et al. 2023), IL‐6 (Dong et al. 2023; Mulati et al. 2021), and IL‐1β (Dong et al. 2023) mRNA levels, microglial activation (ionized calcium‐binding adapter molecule‐1 immunofluorescence intensity) (Dong et al. 2023; Mulati et al. 2021; Tian et al. 2024), and astrocyte activation (glial fibrillary acidic protein (GFAP) immunofluorescence intensity) (Dong et al. 2023; Tian et al. 2024). These parameters are considered as the main components of neuroinflammation (Dong et al. 2023; Mulati et al. 2021; Tian et al. 2024). On the contrary, dietary polyphenols, especially a stilbene resveratrol (RSV), may reduce neuroinflammation, have neuroprotective effects (Rahimifard et al. 2017), and are recommended in the dietary strategies to prevent neurodegenerative diseases (Moussa et al. 2017). Resveratrol reduces plasma levels of IL‐12p70 (Moussa et al. 2017) and protects against depression (Baghaei Naeini, Hassanpour, and Asghari 2023) due to its anti‐inflammatory effects.

Coconut oil (CO) has become a popular dietary fac due to its desirable fatty acid composition (Chatterjee et al. 2020). CO may improve cognitive functions in Alzheimer's patients (de la Rubia Ortí et al. 2018), which is attributed to its primary fatty acid, lauric acid (LA) (Chatterjee et al. 2020; DiNicolantonio and O'Keefe 2017). The mechanisms of the medium‐chain fatty acid (MCFA) LA (12:0) are not well known, and only a few studies investigate its influence on diet‐induced neuroinflammation. LA showed anti‐inflammatory effects in human neuroblastoma cells (Ramya et al. 2022) and attenuated LPS‐induced microglial activation (Nishimura et al. 2018). As a saturated fatty acid (SFA), LA may also trigger inflammatory pathways and lead to pro‐inflammatory cytokine and chemokine production (Rocha et al. 2016). Hence, LA's effects on neuroinflammation are unclear, with limited in vivo studies of its dietary intake.

Recently, the role of cluster of differentiation 36 (CD36) in neuroinflammation as a potential therapeutic target has been investigated (Dobri et al. 2021). CD36 is detected in mouse microglia, and astrocytes are responsible for amyloid clearance, correlated with a pro‐inflammatory response (Dobri et al. 2021). Previous studies have shown that high‐fat (Nergiz‐Unal et al. 2020) and high‐fructose diets (Kisioglu and Nergiz‐Unal 2020; Nergiz‐Unal et al. 2020) influence CD36 expression in various tissues. Further, CD36 can transfer intracellular signals associated with the initiation of inflammation due to increased fat intake (Love‐Gregory and Abumrad 2011). However, the effects of different diets and nutrients on the expression of brain CD36 and related neuroinflammatory pathways are not known.

To conclude, Western diets characterized by high intake of fat and added sugars may not only change and accelerate hallmarks of neurodegenerative diseases but can also be an initiating factor in the development of neurodegenerative diseases (Więckowska‐Gacek et al. 2021). Therefore, this study was designed to investigate the effects of RSV or LA intake on high‐fat/high‐fructose diet (HFFD)‐induced neuroinflammation and with the proposed role of CD36.

## 2 Experimental Section

### 2.1 Animals and Dietary Experimental Design

All the experiments followed the “Animal Research: Reporting in Vivo Experiments (ARRIVE)” guideline for animal care. Thirty‐two male C57BL/6 mice (8 weeks of age, 24–27 g) were purchased from Hacettepe University Experimental Animal Research and Husbandry Unit, Ankara, Turkey. The dietary interventions were carried out in a temperature (22 ± 1°C), humidity (45 ± 5%), and light (12:12 light–dark cycle) controlled room as mice were individually housed. During the study period, one mouse from the HFFD‐RSV group died for unknown reasons.

### 2.2 Dosage Information

**FIGURE 1:**
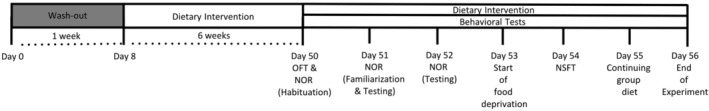
The study design. NOR, Novel object recognition test; NSFT, Novelty suppressed feeding test; OFT. Open field test.

All mice were given free access (*ad libitum*) to chow and drinking water. During the standardization period, all mice received standard chow and drinking water. Then, mice were randomly divided into four dietary groups and fed for 6 weeks (Figure 1). The control group (*n* = 8) received a standard diet (4.4 g fat/100 g, 3.82 kcal/g—10%kcal from fat) (#D12450J, Research Diets) whereas the other three groups HFFD (*n* = 8), HFFD‐RSV (*n* = 7), and HFFD‐LA (*n* = 8) received a high‐fat chow (34.9 g fat/100 g, 5.21 kcal/g—60%kcal from fat) (#D12492, Research Diets) and fructose added drinking water (5% w/v—5 g/100 mL). In addition to their high‐fat, high‐fructose diet, HFFD‐RSV received RSV (7.5 mg/kg/bw, Solgar Inc., New Jersey, USA) (Huang et al. 2018) and HFFD‐LA received LA (750 mg/kg/bw, Merck & Co., New Jersey, USA) (Lekshmi Sheela et al. 2016) as daily oral gavage. RSV and LA solutions were prepared fresh daily in 0.9% NaCl. Thus, the control and HFFD groups also received a daily gavage (0.9% NaCl) without a dietary ingredient. The LA solutions were heated to the melting point of LA (45°C), to enhance solubility, then cooled back to room temperature, and carefully checked for precipitation before the gavage procedure (National Center for Biotechnology Information 2024; Troxler et al. 2023). The daily gavage was performed between 8:00 and 10:00 am. The animal feed used in this study was purchased from Arden Research & Experiment Co., Ltd., Ankara, Turkey. It was prepared according to the National Research Council (NRC) and OpenSource Diets. The composition of the diet is shown in Table 1.

**TABLE 1:** Composition of the diets administrated with feed, water, and gavage during the dietary study.

Diet composition^b^	Experimental diet groups^a^			
	Control	HFFD	HFFD‐RSV	HFFD‐LA
Energy (kcal/g)				
Energy, feed (kcal/g)	3.83	5.21	5.21	5.21
Carbohydrate (% energy)				
Carbohydrate, feed (% energy)	70	20	20	20
Fat (% energy)^c^	10	60	60	60
Protein (% energy)	20	20	20	20
Carbohydrate source (g/100 g chow)				
Corn starch	52.13	—	—	—
Maltodextrin	14.22	16.15	16.15	16.15
Sucrose	—	8.89	8.89	8.89
Cellulose	4.74	6.46	6.46	6.46
Fat source (g/100 g chow)				
Palm oil	4.26	—	—	—
Butter (anhydrous)	—	34.89	34.89	34.89
Protein source (g/100 g chow)				
Casein	18.95	25.85	25.85	25.85
L‐cysteine	0.28	0.39	0.39	0.39
Others (g/100 g chow)				
Mineral mix	0.95	1.29	1.29	1.29
Vitamin mix	0.95	1.29	1.29	1.29
Choline chloride	0.19	—	—	—
Choline bitartrate	—	0.26	0.26	0.26
Dicalcium phosphate	1.23	1.68	1.68	1.68
Calcium carbonate	0.52	0.71	0.71	0.71
Potassium citrate	1.56	2.13	2.13	2.13
Energy (kcal/mL)				
Energy, water (kcal/mL)	—	0.2	0.2	0.2
Carbohydrate (% energy)				
Added fructose, water (% energy)	—	100	100	100
Resveratrol (mg/kg)	—	—	7.5	—
Lauric acid (mg/kg)	—	—	—	750

^a^ Control is the standard chow diet; HFFD is the high‐fat/high‐fructose diet; HFFD‐RSV is the resveratrol added high‐fat/high‐fructose diet; HFFD‐LA is the lauric acid added high‐fat/high‐fructose diet.

^b^ The diet composition was divided into feed, water, and gavage.

^c^ The energy from lauric acid was ignored due to very low contribution.

The human equivalent doses of RSV and LA are 0.6 mg/kg and 60 mg/kg, respectively (Reagan‐Shaw, Nihal, and Ahmad 2008). Rodent studies have used RSV at doses between 4 and 300 mg/kg (Khorshidi et al. 2021). The estimated dietary intake of RSV is reported as only a few mg/day, where red wine contributes to its highest source (approximately 90%) (Pastor et al. 2019). Although the dietary intake of LA is not estimated, it is accepted as one of the highest consumed dietary SFA, contributing to 0.2% energy intake in men and women (Zong et al. 2016). In our study, LA contributed to 3% of the daily energy intake of HFFD‐LA mice.

### 2.3 Assessment of Animal Behavior

The behavioral tests were performed between 10:00 and 17:00 h during the lights‐on phase to avoid the stress of the daily gavage. Between tests, the apparatus and objects were cleaned with 70% ethanol to mask olfactory cues. All the tests were recorded with a camera (Fujifilm X‐A2, Japan), and the behavior of the mouse was analyzed using a commercially available video‐tracking system (ANY‐maze, Stoelting Co., USA).

#### 2.3.1 Open Field Test

Anxiety‐like behavior and locomotor activity were evaluated by the Open Field Test (OFT) (Seibenhener and Wooten 2015). The apparatus consisted of a 40 cm (length) × 40 cm (width) × 30 cm (height) black cardboard‐covered plexiglass box. The activity of each mouse left in the middle of the test box was recorded for 10 min.

#### 2.3.2 Novel Object Recognition Test

The Novel Object Recognition Test (NOR) was performed to test the recognition memory of the mouse's natural preference to explore novel objects conducted in an open field arena. This test was performed in four sessions (habituation and familiarization sessions, test phases 1 and 2) in an open field and lasted for 3 days (Figure 1A). Every session lasted for 10 min. Since the test apparatus is the same as the OFT, the OFT was accepted as the habituation session of NOR. Test phase 1 was performed 1 h later, and test phase 2 was performed 24 h after the familiarization session (Figure 1). Mice were allowed to recognize the objects. Exploration of both the objects obtained from the software was used to calculate the discrimination index (DI) [(time exploring the novel object/total time exploring both objects) × 100] to measure short‐term (1 h) and long‐term (24 h) recognition memory (Antunes and Biala 2012). For exploration, software's setting was a distance of ≥ 2 cm to the object from the mouse's nose. Each video was checked for false calculations of exploration (Antunes and Biala 2012).

#### 2.3.3 Novelty‐Suppressed Feeding Test

This novelty‐suppressed feeding test (NSFT) of animal behavior measures anxiety in a novel environment generated by hunger response (Gomes et al. 2020). The experiment was performed as mentioned (Gomes et al. 2020). The time the animal takes to eat the food pellet in the center was recorded (latency to eat). The cut‐off time was 10 min.

### 2.4 Collection of Blood and Tissues

At the end of the dietary manipulation and behavioral tests, mice (16 weeks of age) were deprived of feed and water for 5 h. They were anesthetized by subcutaneous injection of ketamine (0.1 mg/g body weight) and xylazine (0.02 mg/g body weight). Subsequently, plasma was collected from venous blood and stored at −80°C. Then, the dissected brain tissues were collected, and one hemisphere of each brain was stored in 10% formaldehyde for immunohistochemistry analysis. The other half was cut into two equal pieces (samples for ELISA/flow cytometry and RT‐qPCR), snap‐frozen in liquid nitrogen, and stored at −80°C.

### 2.5 Measurement of Plasma and Brain Inflammatory Mediators

Flow cytometry analysis was performed to analyze the inflammatory mediators (IL‐6, IL‐10, IL12p70, MCP‐1, TNF‐α, and IFN‐γ) in plasma and brain samples, as previously mentioned (Tamer et al. 2020). Before the assay, the brain tissues were lysed using RIPA buffer (Sigma‐Aldrich Corp., USA) in the presence of protease and phosphatase inhibitors (Thermo Fisher Scientific, USA). The amount of protein in lysates was quantified using the BCA assay kit (Bio‐Rad, USA).

### 2.6 Measurement of CD36 and GFAP

Concentrations of plasma and brain tissues of cluster of differentiation 36 (CD36) and GFAP and adipose CD36 were measured using commercially available ELISA kits (Bioassay Technology Laboratory, China), following the manufacturer instructions by using an Absorbance Microplate Reader (ChromMate 4300, Awareness Technology Inc., USA). The brain tissue samples were prepared as previously described in flow cytometry samples.

### 2.7 Gene Expression Analysis of the Brain by RT‐qPCR

The total RNA of the brain tissues was purified by using Trizol (Invitrogen, Carlsbad, CA, USA). After the extraction, the total RNA for each sample was quantified (Berthold Technologies GmbH & Co. KG, Germany). Real‐time PCR of cDNA was performed (Thermo Fisher Scientific, USA) using forward and reverse primer sequences. Data were analyzed using a comparative critical threshold (Ct) method where the amount of target normalized to the amount of endogenous control (β‐actin) relative to the control sample was determined by the 2^−ΔΔCt^ method. The mRNA expressions of TNF‐α and IL‐6 were measured. The forward and reverse primer sequences were as follows (5′–3′): TNF‐α: CCAAAGGGATGAGAAGTTC and GCTACAGGCTTGTCACT, IL‐6: CCTGTCTATACCACTTCAC and GCATCATCGTTGTTCATAC, β‐actin: TGAAGATCAAGATCATTGCT and GAAGGTGGACAGTGAGG. The annealing temperature for TNF‐α, IL‐6, and β‐actin was 61°C, 64°C, and 59°C, respectively. After 40 cycles, the fluorescence value surpassing the threshold value (Ct, Cp, Cq) was used for calculations (Livak and Schmittgen 2001; Willems, Leyns, and Vandesompele 2008).

### 2.8 Immunohistological Procedure for the Brain

At the end of the experiment, brain tissues taken from the mice were fixed in 10% formaldehyde solution. After the tissues were kept in the fixation solution, the water was removed by passing through the tissues with increasing alcohol series. Sections were kept in xylene, and paraffin was blocked. Five μm sections were spread on polylysine‐coated slides. IL‐6 immunohistochemistry was performed for brain tissues using these sections (Karabulut et al. 2020). Sections kept in the oven were passed through xylene and then graded into alcohol series. PBS was used as the washing solution. Sections were treated in a microwave oven with 5% citrate buffer for antigen recovery. Sections kept in hydrogen peroxide were washed with PBS. The procedures after this step were performed using the immunohistochemistry staining kit (LabVision, Ultra Vision Detection System Large Volume Anti‐Polyvalent, ThermoScientific) according to the manufacturer's instructions. Diaminobenzidine (DAB Plus Substrate System, ThermoScientific) was applied to visualize IL‐6 (ab9324, Abcam) immunoreactivities, and the sections were washed with water. Sections counterstained with Gill hematoxylin were rewashed with water and closed by passing through alcohol and then xylene steps. Pictures were taken from the cortex layer of all brain tissues. IL‐6 immunoreactivity was measured using the Image J program for one hundred areas in each group.

### 2.9 Statistical Analysis

The G*Power program was used to calculate the sample size of this study. According to the difference between the highest and lowest average groups, the effect size was calculated as 0.5685. Eight mice per group and a total of 32 mice were calculated to be used in this study as the type I error (α) was accepted as 0.05, and the power of the test (1−β) was accepted as 80%. Statistical tests were performed to analyze differences between all four groups, as previously mentioned (Buyukdere, Gulec, and Akyol 2019). To determine the inter‐relationship of multiple variables and to evaluate the complex effect of the diets, the Principal Component Analysis (PCA) was performed using Python (https://www.python.org/) programming language. Statistical analyses using SPSS version 23 (SPSS, Chicago, IL, USA) considered *p* < 0.05 statistically significant.

## 3 Results

### 3.1 Neuroinflammation Analysis of the Brain

**FIGURE 2:**
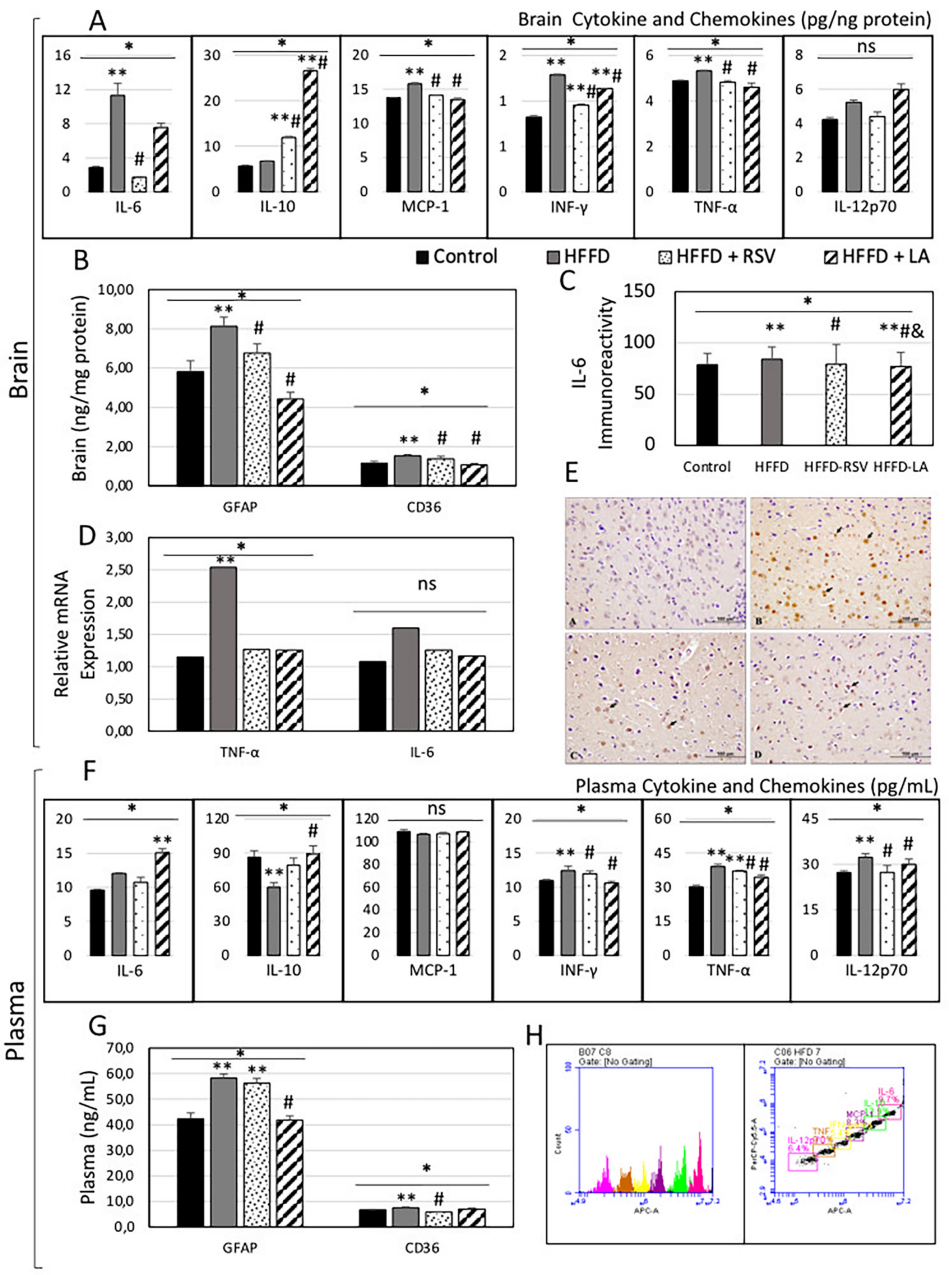
Neuroinflammation and systemic inflammation of the diets. (A) Brain cytokine and chemokine levels; (B) brain GFAP and CD36 levels; (C) graph of brain tissue IL‐6 immunoreactivity results; (D) relative mRNA expression of genes in the brain; (E) brain tissue sections of IL‐6 immunoreactivity (black arrows); (F) plasma cytokine and chemokine levels; (G) plasma GFAP and CD36 levels; (H) representative images of the analysis of cytokines and chemokines by flow cytometry. Mean ± SEM. *p < 0.05 One‐Way ANOVA; **p < 0.05 versus control; #*p* < 0.05 versus HFFD; &*p* < 0.05 versus HFFD‐RSV; CD36, Cluster of differentiation 36; Control, Control diet; GFAP, Glial fibrillary acidic protein; HFFD, High‐fat high‐fructose diet; LA, Lauric acid; NS, Not significant; RSV, Resveratrol.

Brain levels of cytokines and chemokines were analyzed since neuroinflammation is associated with changed levels of cytokines and chemokines. The HFFD increased brain levels of IL‐6, MCP‐1, IFN‐γ, and TNF‐α (*p* < 0.05). HFFD‐RSV reduced brain concentrations of IL‐6, MCP‐1, IFN‐γ, and TNF‐α compared to HFFD (*p* < 0.05). HFFD‐LA reduced brain concentrations of MCP‐1, IFN‐γ, and TNF‐α compared to HFFD (*p* < 0.05). Both HFFD‐RSV and HFFD‐LA diet groups had increased brain IL‐10 levels compared to HFFD and control diets (*p* < 0.05) (Figure 2A).

Brain levels of GFAP and CD36 were analyzed since neuroinflammation is typically characterized by more clusters of microglia (marked by CD36) and activated astrocytes (marked by GFAP). Brain GFAP and CD36 concentrations were significantly higher in the HFFD than in the control diet (*p* < 0.05). The HFFD‐LA and HFFD‐RSV had lower brain GFAP levels than the HFFD (*p* < 0.05) (Figure 2B).

To further detect neuroinflammation in the brain, relative gene expression and immunolabeling for markers of neuroinflammation were examined. HFFD increased brain TNF‐α mRNA expressions compared to the control (*p* < 0.05). Although brain IL‐6 mRNA expressions were not different among the dietary groups, the detected IL‐6 immunoreactivity in brain tissues showed a higher value due to the HFFD than the control diet (*p* = 0.0001). The HFFD‐RSV and HFFD‐LA diets showed lower brain IL‐6 immunoreactivity compared to HFFD (*p* = 0.0001). The HFFD‐LA diet showed lower IL‐6 brain immunoreactivity compared to HFFD‐RSV and the control diet (*p* = 0.0001) (Figure 2C–E).

### 3.2 Systemic Inflammation Analysis of Plasma

Systemic inflammation was detected by analyzing plasma levels of cytokines and chemokines. Plasma levels of IFN‐γ, TNF‐α, and IL‐12p70 increased, whereas IL‐10 decreased in the HFFD compared to the control diet (*p* < 0.05). The HFFD‐RSV decreased plasma levels of IFN‐γ, TNF‐α, and IL‐12p70 compared to HFFD and increased plasma TNF‐α compared to the control diet (*p* < 0.05). The HFFD‐LA decreased plasma levels of IFN‐γ, TNF‐α, and IL‐12p70 and increased plasma IL‐6 and IL‐10 compared to HFFD or control diet (*p* < 0.05) (Figure 2F,H).

Plasma levels of an astrocyte marker GFAP and novel marker CD36 were analyzed to distinguish the effect of the diets. Plasma GFAP levels were significantly higher in the HFFD and HFFD‐RSV than in the control diet (*p* < 0.05). The HFFD‐LA lowered plasma GFAP levels compared to HFFD (*p* < 0.05). Plasma CD36 levels were high in the HFFD compared to the control diet and lower in the HFFD‐RSV than the HFFD (*p* < 0.05) (Figure 2G).

### 3.3 Anxiety‐Like Behavior, Locomotor Activity, and Recognition Memory

**FIGURE 3:**
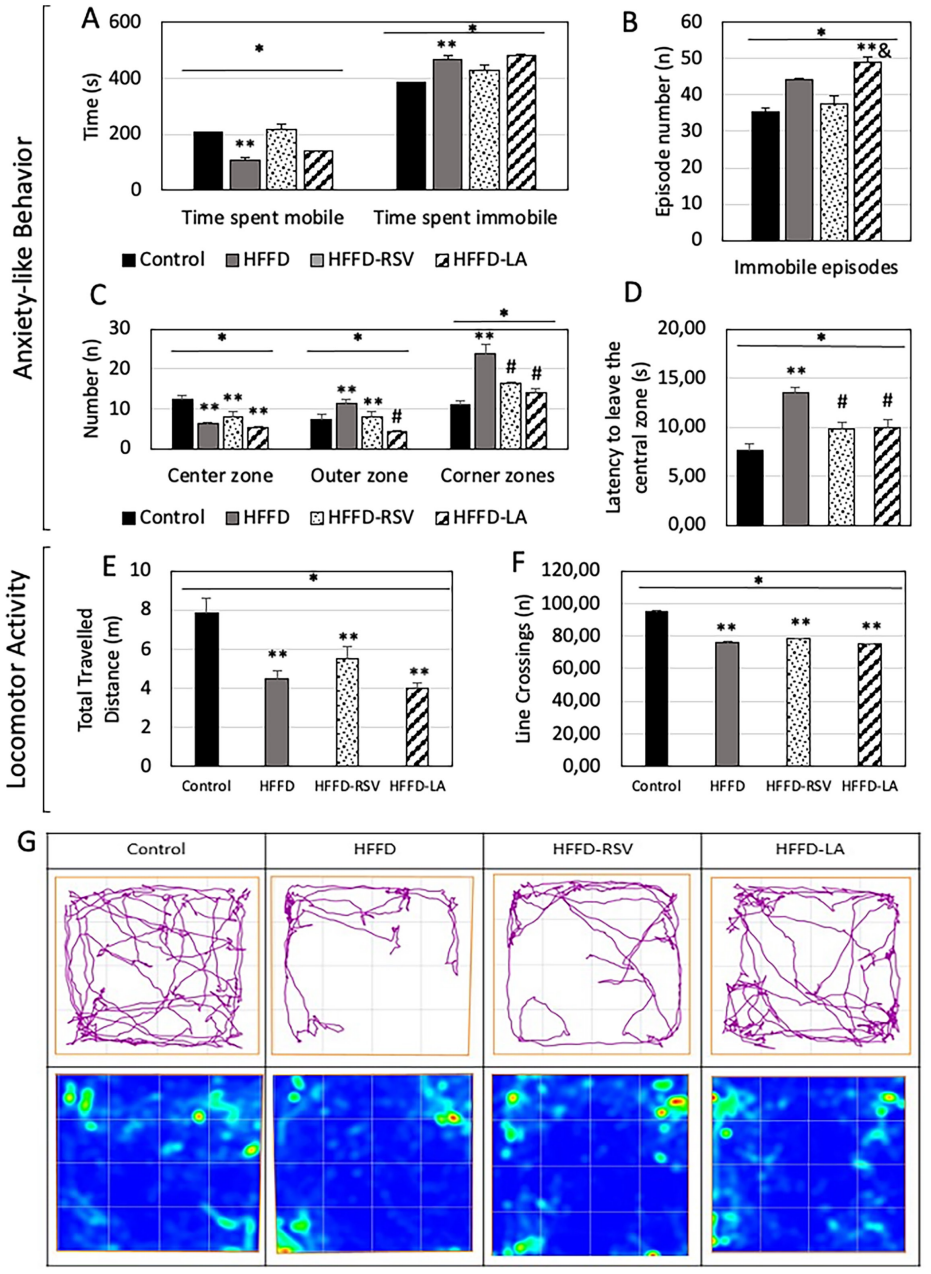
Anxiety‐like behavior and locomotor activity measures of the open field test in mice. (A) Time spent mobile and immobile; (B) total immobile episodes; (C) times entered the center, outer, and corner zones; (D) latency to leave the central zone; (E) total distance traveled; (F) Line crossings of the 16 square shaped arena; (G) representative trace images and heat maps of movement in each diet group. Mean ± SEM. *p < 0.05 One‐Way ANOVA; **p < 0.05 versus control; #*p* < 0.05 versus HFFD; &*p* < 0.05 versus HFFD‐RSV; Control, Control diet; HFFD, High‐fat high‐fructose diet; LA, Lauric acid; NS, Not significant; RSV, Resveratrol.

Behavioral tests were analyzed for further evaluation of the dietary manipulations. Compared to the control diet, HFFD showed lower time spent mobile, higher time spent immobile, lower number of entries to the center zone, and high number of entries to the outer and corner zones, indicating anxiety‐like behavior (*p* < 0.05). The HFFD‐RSV showed a lower number of entries to the center zone and a higher number of entries to the outer zone compared to the control diet; however, there was a lower number of entries to the corner zones than HFFD (*p* < 0.05). The HFFD‐LA showed a lower number of entries to the center zone compared to the control diet; however, a lower number of entries to the outer and corner zones compared to HFFD (*p* < 0.05). Additionally, the number of immobile episodes of the HFFD‐LA was higher than the control diet and HFFD‐RSV, indicating anxiety‐like behavior (*p* < 0.05). The latency to leave the central zone of the OFT was higher in the HFFD compared to the control diet, indicating anxiety‐like behavior, whereas HFFD‐RSV and HFFD‐LA showed lower values compared to HFFD (*p* < 0.05). Both the total traveled distance and the number of line crossings of OFT were significantly lower in the HFFD, HFFD‐RSV, and HFFD‐LA than in the control diet (*p* < 0.05) (Figure 3A–G).

**FIGURE 4:**
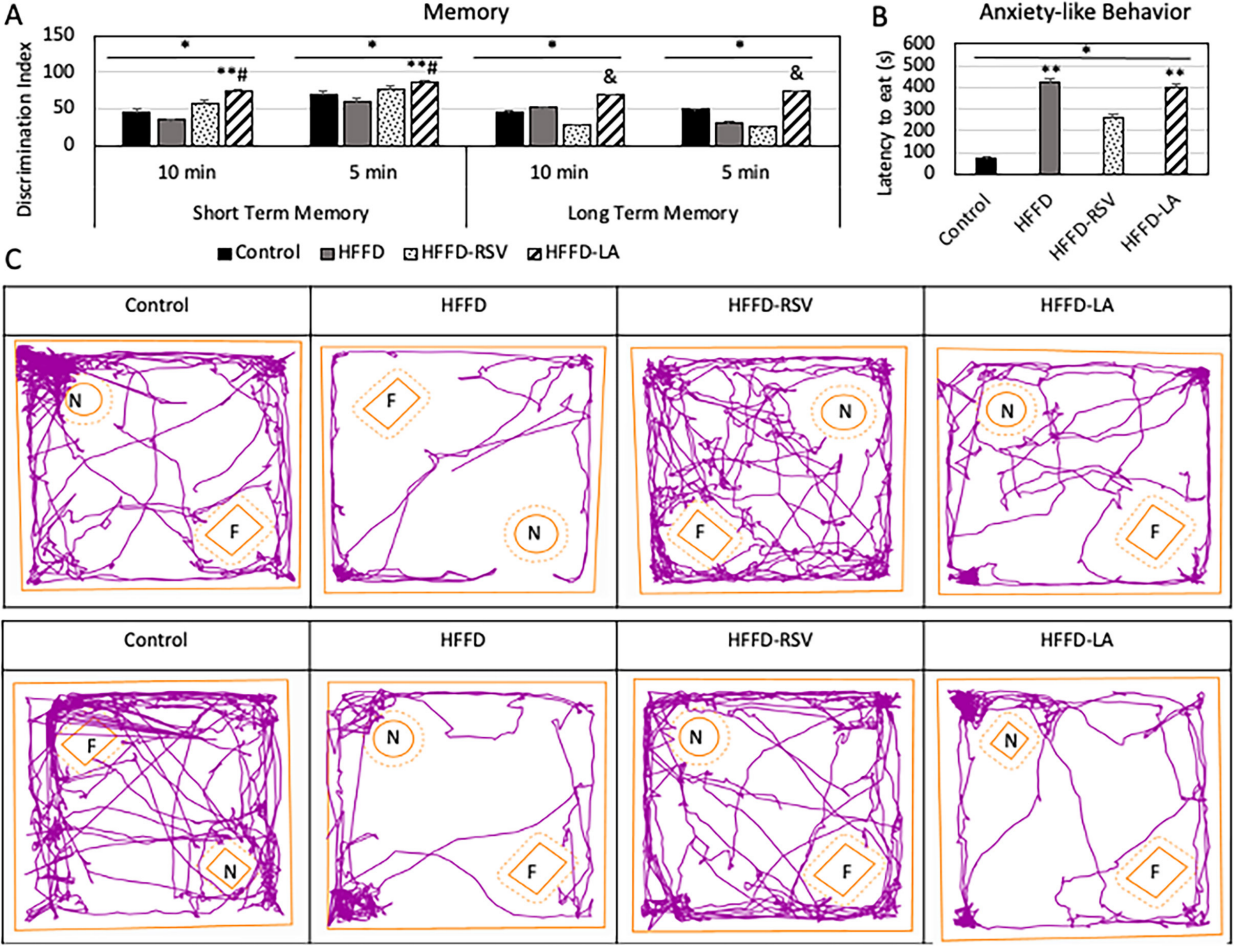
Anxiety‐like behavior and recognition memory measured by behavioral tests in mice. (A) Discrimination index values of the Novel Object Recognition Test; (B) latency to eat measured by the Novelty Suppressed Feeding Test; (C) representative trace images of the movement in each diet group of the Novel Object Recognition Test. Mean ± SEM. *p < 0.05 One‐Way ANOVA; **p < 0.05 versus control; #*p* < 0.05 versus HFFD; &*p* < 0.05 versus HFFD‐RSV; Control, Control diet; F, Familiar object; HFFD, High‐fat high‐fructose diet; LA, Lauric acid; N, Novel object; NS, Not significant; RSV, Resveratrol.

Short‐term (1 h) and long‐term (24 h) recognition memory with two different video lengths (10 min and 5 min) was evaluated. The DI of short‐term memory was found to be higher in the HFFD‐LA compared to the HFFD and control diet (*p* < 0.05). Further, higher DI values in long‐term memory were shown in HFFD‐LA compared to HFFD‐RSV (*p* < 0.05) (Figure 4A,C). Additionally, NSFT showed an increase in feeding latency by the HFFD and HFFD‐LA compared to the control diet, indicating anxiety‐like behavior (*p* < 0.05) (Figure 4B).

### 3.4 PCA Analysis and the Relationship Between Variables

We analyzed the data of this study using PCA to identify the effects of the diets and to investigate the relationship between different variables. All the data for the dietary groups were separated according to the principal components (PC) 1 and 2, which showed that the plasma IL‐6 and plasma CD36 were the primary separators of all the dietary groups. Brain CD36 and latency to eat in NSFT were the primary separators of HFFD from the control diet. Brain GFAP was the primary separator of HFFD‐RSV, whereas plasma IL‐12p70 was the primary separator of HFFD‐LA from HFFD. In addition, brain IL‐6 mRNA expression and DI values were the primary separators of HFFD‐LA from HFFD‐RSV. PCA revealed a strong positive correlation between brain CD36 levels and brain IFN‐γ, IL‐6, and IL‐12p70, whereas plasma CD36 was negatively correlated with the total traveled distance in OFT. Brain GFAP concentrations were strongly correlated positively with time spent immobile in OFT, whereas plasma GFAP levels were positively correlated with immobile episodes.

**FIGURE 5:**
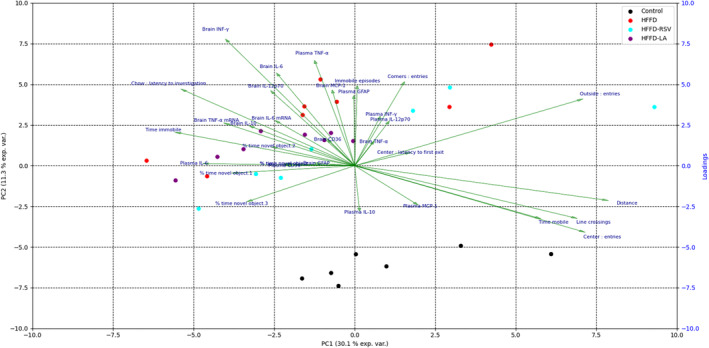
Principal Component Analysis (PCA). Biplot of the results shown with all the four dietary groups. Points represent the projection of all mice on the first two principal components (PCs). Green arrows show the relationship between different variables. The angle between the arrows is inversely proportional to the magnitude of the correlation; 90° means no correlation, and 180° means correlation −1. PCA loading values showing arrows are shown in 10×.

Further, plasma IL‐10 was negatively correlated with plasma GFAP, plasma IFN‐γ, and brain TNF‐α. Additionally, the DI values in NOR were positively correlated with brain IL‐10, whereas they negatively correlated with plasma CD36 and plasma IL‐6. Latency to eat in NSFT was positively correlated with brain TNF‐α and plasma MCP‐1 levels (Figure 5).

## 4 Discussion

The molecular mechanisms involved in the connection between diet and neurodegenerative disease development are increasing targets of interest by the scientific community (Nakandakari et al. 2019). The literature points out that Western‐type diets that are high in energy, added sugars (fructose or sucrose), and fats are found to be associated with neuroinflammation, the hallmark of neurodegenerative diseases (Ho et al. 2020; Li et al. 2019). Potential dietary factors such as polyphenols may be used to reduce neuroinflammation (Rahimifard et al. 2017), and potential foods such as CO are of interest in neurodegenerative diseases (Chatterjee et al. 2020; de la Rubia Ortí et al. 2018). Therefore, this study investigated the effects of LA, the major fatty acid in CO, and RSV intake on HFFD‐induced neuroinflammation.

Systemic inflammation may lead to neuroinflammation or vice versa, which is still a topic under investigation (Woo et al. 2022). Our study successfully demonstrated that HFFD induced neuroinflammation and systemic inflammation. Correspondingly, high fat (Alghamdi 2021; Nakandakari et al. 2019; Tamer et al. 2020; Yu et al. 2019), saturated fat (Tamer et al. 2020), and fructose (Gomes et al. 2020; Ho et al. 2020; Li et al. 2019; Tamer et al. 2020; Yu et al. 2019) intake was associated with higher levels of IL‐6 (Alghamdi 2021; Gomes et al. 2020; Ho et al. 2020; Li et al. 2019; Tamer et al. 2020; Yu et al. 2019), IL‐1β (Alghamdi 2021; Ho et al. 2020; Li et al. 2019; Nakandakari et al. 2019; Yu et al. 2019), TNF‐α (Alghamdi 2021; Gomes et al. 2020; Ho et al. 2020; Li et al. 2019; Nakandakari et al. 2019; Tamer et al. 2020; Yu et al. 2019), and MCP‐1 (Tamer et al. 2020) along with lower levels of IL‐10 (Tamer et al. 2020) in the plasma and brain of mice. This may be due to activated microglia and astrocytes (Rahimifard et al. 2017) as high‐fat (Alghamdi 2021; de Paula et al. 2021; Nakandakari et al. 2019) and high‐fructose diets (Ho et al. 2020; Li et al. 2019) led to astrocytic activation (GFAP) in the brain (Li et al. 2019; Yu et al. 2019). Moreover, plasma GFAP has been associated with brain amyloid pathology (Pereira et al. 2021) and cognitive impairment (Cicognola et al. 2021) in humans, but it is not widely studied, and its dietary response has not been investigated. Further, a novel biomarker, CD36, was investigated as a potential underlying mechanism. Limited evidence indicated increased brain CD36 expression due to high‐fat diets (17%–60%kcal fat), associated with neuroinflammation (Moser, Uchoa, and Pike 2018; Treviño et al. 2022). CD36 induces microglial activation into a pro‐inflammatory state (Dobri et al. 2021), which may explain the positively correlated brain CD36 levels with brain levels of IFN‐γ, IL‐6, and IL‐12p70. Thus, the present study provides novel results by increasing brain and plasma GFAP and CD36 levels in mice due to HFFD.

Neuroinflammation may lead to impaired cognition and memory, also induced by diets high in fat (Alghamdi 2021; Yu et al. 2019) and fructose (Sangüesa et al. 2018; Yu et al. 2019). Although HFFD did not lead to cognitive impairment in the present study, DI values were positively correlated with brain IL‐10 and negatively correlated with plasma CD36 and IL‐6. Moreover, literature has pointed out that diets high in fat (Alghamdi 2021; Gancheva, Galunska, and Zhelyazkova‐Savova 2017; Nakajima et al. 2020) and fructose (Gancheva, Galunska, and Zhelyazkova‐Savova 2017; Nakajima et al. 2020) may induce anxiety (Alghamdi 2021; Gancheva, Galunska, and Zhelyazkova‐Savova 2017; Kloster et al. 2021; Nakajima et al. 2020) associated with neuroinflammation (Gomes et al. 2020), as shown by many PCA correlations in the present study. Further, the present study revealed that low plasma CD36 may indicate increased recognition memory and reduced anxiety‐like behavior. Although there are limited studies, CD36 may be associated with impaired memory and learning ability (Zhang et al. 2018) instead of anxiety (Abumrad et al. 2005).

CO is of interest as a potential food against neurodegenerative diseases (Chatterjee et al. 2020; de la Rubia Ortí et al. 2018); however, its primary fatty acid, LA, is poorly investigated (Chatterjee et al. 2020; Fosdick et al. 2022; Kong et al. 2021). Studies have reported inhibited activation of T and B lymphocytes (Fosdick et al. 2022), reduced IL‐1β and TNF‐α levels in serum (Kong et al. 2021), and suppressed IL‐1β, TNF‐α, and IL‐6 production in LPS‐induced microglia (Nishimura et al. 2018) due to LA (Huang et al. 2014; Nishimura et al. 2018; Ramya et al. 2022) and glycerol monolaurate (monoglyceride form of LA) (Fosdick et al. 2022; Zhao et al. 2019) as anti‐inflammatory effects. On the contrary, as an SFA, LA increased the release of TNF‐α, IL‐6, and IL‐1β from cultured astrocytes (Gupta et al. 2012) and induced a cytokine storm by hyperactivating IL‐6 and TNF‐α in a human‐derived cell line (Ramya et al. 2022) due to the activation of TLRs (Nakandakari et al. 2019; Rocha et al. 2016; Wang et al. 2012). In addition, our study also provides data on the anti‐inflammatory effects of LA by alleviating HFFD‐induced neuroinflammation and systemic inflammation.

Suppressing the hyperactivation of microglia and astrocytes is an essential factor for slowing the progression of neurodegenerative diseases (Nishimura et al. 2018); thus, lower brain GFAP and CD36 concentrations may indicate suppressed neuroinflammation. To our knowledge, no studies have investigated the effects of LA on GFAP and brain CD36, which is the superiority of this study. Virgin CO in rats led to reduced brain GFAP immunoexpression (Shehata et al. 2020), suggesting that CO‐derived LA, compared to other MCFA, can be sustained at a high concentration in the blood before metabolized into ketone bodies (Nishimura et al. 2018). Thus LA may potentially induce astrocytic rather than hepatocyte ketogenesis (Nonaka et al. 2016). Further, this study may show that the increased resolution of neuroinflammation/systemic inflammation (IL‐10) due to HFFD‐LA might alleviate neuroinflammation and reduce plasma GFAP levels. Additionally, studies investigating monolaurate (Zhao et al. 2020) or CO (Borcherding et al. 2022; Lee et al. 2018) showed reduced levels of CD36 in different tissues (Borcherding et al. 2022; Lee et al. 2018; Zhao et al. 2020) either alone or supplemented to an HFD (39%–45%kcal fat) as protective effects against fatty liver (Lee et al. 2018; Zhao et al. 2020) and obesity (Borcherding et al. 2022). LA also mitigated amyloid β‐induced phagocytosis proposedly by a G‐protein‐coupled receptor‐40, responsive to fatty acids (Nishimura et al. 2018). Thus, the response of CD36 to different fatty acids is essential for the treatments against neurodegenerative diseases.

Following its anti‐neuroinflammatory effects, LA supplementation alleviated anxiety‐like behavior and improved memory. Nevertheless, no studies have investigated the effects of dietary LA intake on behavior and cognition. Virgin CO attenuated the increased anxiety‐related effects of L‐dopa (Shehata et al. 2020), yet a CO consisting of HFD (60% fat) did not change cognitive function (Alghamdi 2021). Interestingly, a CO‐enriched Mediterranean diet for Alzheimer's patients improved cognitive functions (de la Rubia Ortí et al. 2018). On the contrary, LA may also induce anxiety‐like behavior since it is an SFA (Moon et al. 2014), as seen in some of our results. To conclude, the lipid composition of the diet is essential and may be involved in the pathophysiology of behavioral changes by altering the brain (Nakajima et al. 2020). Although LA might have conflicting results in anxiety‐like behavior, its anti‐inflammatory and anti‐neuroinflammatory effects may improve cognition.

Accumulating evidence showed that RSV may have immunomodulatory effects (Trusov et al. 2021) and may inhibit neuroinflammation (Lu et al. 2010) by the downregulation of GFAP in activated astrocytes and microglia (Yang et al. 2016). These anti‐neuroinflammatory effects can be attributable to modulating levels of cytokines (Lu et al. 2010; Moussa et al. 2017) and reducing the overproduction of pro‐inflammatory cytokines (Lu et al. 2010), TNF‐α (Lu et al. 2010; Yang et al. 2016), IL‐1β (Lu et al. 2010; Yang et al. 2016), IL‐6 (Lu et al. 2010), and MCP‐1 (Lu et al. 2010) in microglia and astrocytes. Moreover, RSV supplementation (7.5–15.6 mg/kg/bw) alone (Gómez‐Zorita et al. 2013), to HFFD (42%kcal fat, 20% w/v fructose) (Trusov et al. 2021) or to mild–moderate AD patients (Moussa et al. 2017) showed increased plasma IL‐10 (Trusov et al. 2021) and decreased serum/plasma TNF‐α, MCP‐1 (Gómez‐Zorita et al. 2013), IL‐12p40, and IL‐12p70 (Moussa et al. 2017) as indicators of reduced systemic inflammation (Gómez‐Zorita et al. 2013; Moussa et al. 2017; Trusov et al. 2021). Hence, the present study likely showed that RSV supplementation alleviated the neuroinflammatory and systemic inflammatory response induced by HFFD. Further, as previously mentioned, lower CD36 concentrations may indicate suppressed neuroinflammation (Nishimura et al. 2018); thus, RSV supplementation may modulate HFFD‐induced neuroinflammation and systemic inflammation by reducing brain and plasma CD36 concentrations, as novel data. Additionally, lower levels of plasma CD36 compared to HFFD‐LA may be due to higher cellular uptake of LA (Nonaka et al. 2016).

The mentioned anti‐inflammatory response of RSV may protect against neuroinflammation‐associated cognitive decline (Moussa et al. 2017) and anxiety‐like behavior (Baghaei Naeini, Hassanpour, and Asghari 2023). This study showed that RSV supplementation may alleviate anxiety‐like behavior induced by HFFD, also shown by previous studies of RSV (15.6 mg/kg/bw) supplemented HFFD (42%kcal fat, 20% w/v fructose)‐fed mice (Baghaei Naeini, Hassanpour, and Asghari 2023; Trusov et al. 2021). Moreover, RSV (50–100 mg/kg) supplementation alleviated reduced memory in NOR induced by a HFD (60% kcal fat), which may be associated with decreased TNF‐α brain levels in mice (Singh and Bodakhe 2022); however, it was shown by a higher daily dosage compared to the present study.

## 5 Conclusion

This study highlights the damaging effects of the consumption of HFFD on neuroinflammation, systemic inflammation, and anxiety‐like behavior in mice. RSV and LA supplementation may alleviate HFFD‐induced neuroinflammation, systemic inflammation, and anxiety. Additionally, LA has improved recognition memory. The metabolism of dietary LA, its potential anti‐inflammatory effects, interaction with other components in CO, and in vivo effects in humans need to be thoroughly investigated. Hence, this study points out the need for intervention strategies in the daily diet to reduce the development or progression of neuroinflammation‐associated neurodegenerative diseases. Further, CD36's role in the central nervous system emphasizes its importance; however, the impact of its dietary regulation and clinical outcomes still need to be entirely understood. This study provides novel preclinical data on brain tissue CD36 associated with diet‐induced neuroinflammation.

## Author Contributions

**Betul Kisioglu:** conceptualization (lead), investigation (lead), methodology (lead), project administration (lead), visualization (lead), writing – original draft (lead), writing – review and editing (lead). **Esra Onal:** investigation (equal). **Derya Karabulut:** investigation (equal). **Ilyas Onbasilar:** methodology (equal). **Asli Akyol:** conceptualization (equal), methodology (equal), project administration (equal), supervision (lead), visualization (equal), writing – review and editing (equal).

## Ethics Statement

All animals received ethical and human care within the provisions of the “National Ministry of Food, Agriculture, and Livestock Regulations on the Protection and Welfare of Animals Used for Experimental and Other Scientific Purposes” and Institutional guidelines. Experiments were approved by the Regional Animal Ethics Committee of Hacettepe University, Ankara, Turkey (IRB Number: 2020/10‐09).

## Conflicts of Interest

The authors declare no competing financial interest or personal relationship with this study. Preliminary data of this study was presented as an oral presentation at The Biochemistry Global Summit, 25th IUBMB Congress, 46th FEBS Congress, 15th PABMB Congress. The abstracts were published in (2022), Talks. *FEBS Open Bio*, 12: 2‐66. https://doi.org/10.1002/2211‐5463.13442.

## Data Availability

The data that support the findings of this study are available on reasonable request from the corresponding author.
